# The brain–bone–gut axis: a microbial bridge underlying multisystem comorbidities

**DOI:** 10.3389/fendo.2026.1755305

**Published:** 2026-02-26

**Authors:** Xingli Xu, Qinghan Ma, Peijie You, Jiong Wu

**Affiliations:** 1 Department of Coloproctology, Yueyang Hospital of Integrated Traditional Chinese and Western Medicine, Shanghai University of Traditional Chinese Medicine, Shanghai, China; 2 Department of Orthopedics and Traumatology, Suzhou Traditional Chinese Medicine (TCM) Hospital Affiliated to Nanjing University of Chinese Medicine, Suzhou, China

**Keywords:** brain–bone–gut axis, gut microbiota, inflammatory bowel disease, neuroimmune regulation, neuroinflammation, osteoporosis, short-chain fatty acids

## Abstract

Multi-axis interactions among the skeletal system, immune system, and gut microbiota (GM) have become a prominent focus of interdisciplinary research. The brain–bone–gut axis, proposed in recent years, provides an integrative physiological framework describing a bidirectional regulatory network linking the central nervous system, bone metabolism, and the GM via neural, endocrine, and immune pathways, thereby offering a unified perspective on multi-organ comorbidities. This article systematically examines the interconnections and synergistic effects across three core pathways within this framework: the brain–bone axis, the gut–bone axis, and the gut–brain axis. It further emphasizes immune-inflammatory processes as a central hub that connects gut dysbiosis with bone metabolic disturbances and alterations in brain function. On this basis, we propose an integrated approach that combines microecological interventions with nutritional and exercise management to improve gut homeostasis, preserve skeletal health, and support brain function, with the overarching aim of generating coordinated benefits across organ systems.

## 1 Introduction

The brain–bone–gut axis has emerged as a novel interdisciplinary paradigm aimed at elucidating the complex interconnected networks formed through biochemical, neural, and endocrine pathways linking the brain, skeletal system, and gut microbiota (GM). These three systems not only collaborate to maintain physiological homeostasis under static conditions but also play pivotal roles in the onset, progression, and outcomes of various chronic diseases. In recent years, the convergence of gut microbial ecology, bone metabolism biology, and neuroimmunoendocrinology has propelled this research field to the forefront of studies on osteoporosis, neurodegeneration, and neuropsychiatric disorders. First, the brain influences bone metabolism through neurotransmitters, neurotrophic factors, and endocrine hormones. For instance, within the hypothalamic–pituitary–bone axis, leptin not only crosses the blood–brain barrier (BBB) to regulate central neural circuits but also modulates osteoblast and osteoclast activity via sympathetic neural pathways, thereby contributing to the dynamic balance of bone remodeling (1). In addition, recent work focusing specifically on the “brain–bone axis” has demonstrated that the CNS regulates bone mass and microarchitecture through neuro-skeletal remodeling pathways, including sympathetic and vagal innervation, neuropeptide Y (NPY), and calcitonin gene–related peptide (CGRP) signaling (2). Second, the GM exerts profound effects on both skeletal and neural functions through its metabolites, regulation of mineral absorption, and maintenance of immune homeostasis (3). Short-chain fatty acids (SCFAs)—including acetate, propionate, and butyrate—produced from microbial fermentation of indigestible dietary fibers have been shown to suppress osteoclastogenesis and promote osteoblast differentiation, thereby preserving bone mineral density (BMD) (4). The GM further modulates the intestinal absorption and metabolism of minerals such as calcium, magnesium, and phosphorus, thereby influencing bone mineralization and mechanical strength (5). Within the gut–brain axis (GBA), microbial metabolites also act through neural, immune, and endocrine pathways to affect brain function and behavior; for example, SCFAs can regulate microglial activation, BBB permeability, and neuroinflammation (6). Importantly, the interactions among these three systems are not unidirectional but instead reflect dynamic bidirectional communication and feedback mechanisms. Psychological stress, anxiety, and chronic activation of the hypothalamic–pituitary–adrenal axis (HPA axis) can reshape gut microbial composition—often reducing SCFA-producing taxa—while promoting neuroinflammation and enhancing bone resorption (7). Conversely, disturbances in bone metabolism (such as osteoporosis) may influence central nervous system function through alterations in the bone marrow microenvironment and bone–neural regulatory circuits. Based on these observations, extended frameworks such as the “gut–bone axis + X” and “brain–bone axis” have been proposed to more comprehensively describe these multisystem interactions (8). Rapid advances in this field have not only provided new etiological perspectives for osteoporosis, neurodegenerative diseases, and psychiatric disorders but have also offered promising strategies for simultaneously improving skeletal and neural function through modulation of the GM. Potential approaches include supplementation with probiotics or prebiotics, high-fiber dietary interventions, and even fecal microbiota transplantation, all aimed at restoring microbial balance to jointly regulate bone metabolism and brain function (9).

**Figure 1:**
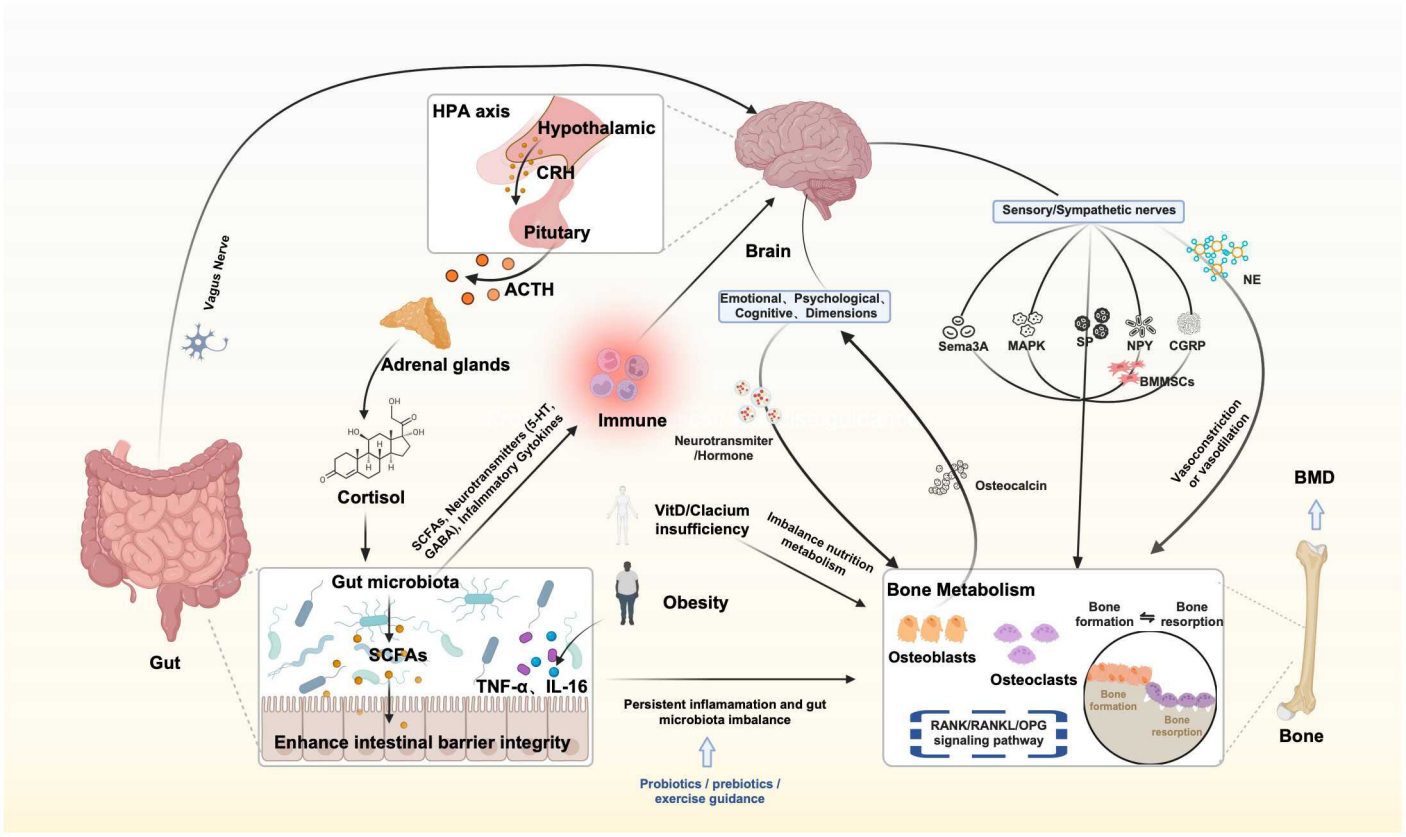
Mechanistic framework of the brain–bone–gut axis.

Accumulating evidence indicates that patients with intestinal diseases carry a substantial multisystem comorbidity burden involving the gut–bone–brain axis. A meta-analysis reported that, among patients with inflammatory bowel disease (IBD), the pooled prevalence of osteoporosis and osteopenia is approximately 12.2% and 31.5%, respectively, and that the odds of osteoporosis are significantly higher than in control populations (OR = 1.64) (10). In parallel, patients with IBD have an increased burden of anxiety and depressive symptoms, which appears to be more pronounced during periods of active disease (11). With respect to cognition, additional analyses suggest that IBD is associated with deficits in multiple domains, including attention, executive function, and working memory (12). Evidence for brain–bone comorbidity further indicates that depression is associated with an elevated risk of fractures and osteoporosis, and that cognitive impairment is likewise associated with a higher risk of osteoporosis (RR = 1.56) (13–15). In summary, maintaining a healthy gut microbiome, optimizing neuro-skeletal-metabolic pathways, and reinforcing the synergistic homeostasis across the brain, bone, and gut systems constitute important directions for future research and clinical intervention. This review is organized around three core components—the brain–bone axis, the bone–gut axis, and the GBA—to synthesize evidence on their bidirectional pathways and to elucidate the interactive, integrative mechanisms that underpin the brain–bone–gut axis (As shown in **FIGURE 1**). By integrating recent advances, we aim to provide a coherent theoretical framework and practical implications for research in orthopedics, neurology, and gut microbiome science.

## 2 Interactions between the brain and bone

The nervous system and bone metabolism are intricately interconnected through highly complex and multilayered regulatory networks that influence both skeletal health and neural function (2). Increasing evidence highlights the central role of the nervous system in the regulation of bone remodeling, demonstrating that neural signaling pathways are indispensable for maintaining bone mass homeostasis and promoting bone tissue regeneration (16). This bidirectional regulation involves a diverse array of neurotransmitters, neuropeptides, and signaling cascades, which collectively mediate communication between neural circuits and skeletal cells. Moreover, emerging research indicates that the skeletal system also exerts endocrine feedback effects on the brain, underscoring the reciprocal nature of the “neuro-skeletal interface” (17).

Sensory and sympathetic nerves exhibit particularly prominent roles in regulating bone metabolism. They release specific neuropeptides that directly modulate the activity of bone cells. For example, substance P (SP), CGRP, and NPY have all been identified as key modulators of skeletal remodeling. NPY not only participates in bone turnover but may also contribute to cartilage degradation; CGRP enhances osteoblast differentiation while inhibiting osteoclast activity, thereby promoting bone formation and supporting BMD (18, 19). SP is additionally involved in neuroinflammation and skeletal repair.

The sympathetic nervous system regulates bone perfusion and remodeling by releasing norepinephrine (NE), which controls vasoconstriction and vasodilation within bone tissue. Chronic stress, however, leads to excessive sympathetic activation, resulting in increased bone resorption and reduced bone formation, ultimately contributing to osteoporosis (20, 21). Evidence also suggests that sympathetic nerves regulate the bone marrow microenvironment, thereby indirectly shaping the dynamic equilibrium of bone metabolism.

The concept of the “bone–brain axis” further emphasizes the reciprocal communication between the nervous system and skeletal tissue. Bone is not merely a target organ for neural signals but also produces endocrine factors that exert feedback effects on the brain. Osteocalcin, secreted by osteoblasts, has been shown to be closely associated with cognition and emotional regulation, suggesting that bone-derived signals may profoundly influence neural health (22, 23). Studies in mouse models demonstrate that osteocalcin contributes to learning, memory, emotional stability, and anxiolytic behavior (24). Conversely, psychological stress and emotional disturbances alter neurotransmitter and hormonal profiles, thereby modulating bone metabolism and influencing bone density and structural integrity (25).

At the molecular level, several critical signaling pathways have been identified as mediators of neural regulation of bone cell function. The Wnt/β-catenin and p38 MAPK pathways constitute major molecular mechanisms underlying neuro-skeletal interactions (19). CGRP activates these pathways to promote the osteogenic differentiation of bone marrow mesenchymal stem cells (BMMSCs), enhancing bone formation and mitigating age-related bone loss (19, 21). Semaphorin 3A (Sema3A), an important regulator of sensory nerve function, also promotes osteoblast differentiation and participates in bone remodeling (26, 27). Its signaling is essential for skeletal regeneration and resistance to bone aging (28).

Understanding neuro-skeletal interactions not only deepens the theoretical foundation of bone metabolism regulation but also offers novel therapeutic opportunities for skeletal disorders. Targeting specific neuropeptides or signaling pathways—such as CGRP or NPY receptor antagonists—has been proposed as a potential strategy for enhancing bone formation, improving fracture healing, and managing osteoporosis (25, 29–31).

Therefore, neuro-skeletal communication should be regarded as a dynamic, bidirectional, and highly integrated regulatory network essential for maintaining skeletal integrity and preventing disease. From a translational standpoint, the bone–brain axis is increasingly viewed as a tractable therapeutic target. For example, augmenting CGRP signaling—such as through CGRP analogs or agonists—may promote osteogenesis and improve fracture healing, whereas inhibiting NPY signaling (e.g., via Y1 receptor antagonists) may mitigate bone loss by enhancing osteoblast activity (32). Pending further validation of dosing, safety, and delivery strategies, these neuromodulatory approaches could complement established antiresorptive and anabolic therapies and support novel combination regimens for osteoporosis prevention and treatment, as well as for bone repair. Future research should further elucidate the multilayered mechanisms linking neural signals and bone metabolism and explore how neuro-skeletal interactions contribute to chronic conditions such as osteoporosis, depression, and aging. Such insights may help identify new targets for early diagnosis, personalized intervention, and therapeutic development.

## 3 Interactions between bone and the gut

The interactions between bone metabolism and the gut microenvironment involve highly complex and multilayered regulatory mechanisms, which are particularly evident in patients with IBD. IBD, comprising primarily Crohn’s disease (CD) and ulcerative colitis (UC), is characterized by chronic inflammation of the gastrointestinal tract and is frequently accompanied by various extraintestinal manifestations, among which pronounced bone loss is one of the most common complications. In recent years, accumulating evidence has shown that systemic inflammatory responses in IBD exert detrimental effects on bone metabolism, primarily through the release of pro-inflammatory cytokines that disrupt the balance between bone resorption and bone formation (33).

### 3.1 Inflammation-mediated alterations in bone metabolism

In patients with IBD, chronic intestinal inflammation activates immune cells and induces the release of pro-inflammatory cytokines—such as tumor necrosis factor-α (TNF-α) and interleukin-6 (IL-6)—which directly promote osteoclastogenesis and accelerate bone resorption, ultimately leading to reduced BMD (34, 35). These cytokines not only inhibit osteoblast function but also exacerbate osteoporosis by enhancing bone resorption while simultaneously suppressing bone formation. Notably, the molecular basis of inflammatory bone loss may extend beyond classical cytokine-mediated pathways. Emerging evidence implicates regulated cell-death programs, particularly ferroptosis, as a potential mechanistic link between inflammatory stress and dysregulated bone metabolism. In a glucocorticoid-induced osteoporosis model, Yang F et al. (36) showed that dexamethasone induces ferroptosis in BMMSCs and compromises their osteogenic differentiation capacity. Mechanistically, pharmacologic inhibition of ferroptosis via activation of the HIF-1α/GPX4 axis improved bone mass and microarchitectural parameters, whereas genetic ablation of HIF-1α eliminated these protective effects, supporting ferroptosis-driven osteogenic dysfunction as a contributory pathway to bone loss. On this basis, it is plausible that under the persistent inflammatory and oxidative stress characteristic of IBD, ferroptosis and related cell-death programs similarly impair osteoblast-lineage function and exacerbate bone loss. Collectively, these findings position ferroptosis as a complementary mechanistic explanation and a potential therapeutic target in inflammatory osteoporosis.

### 3.2 Gut microbiota and bone metabolism

The contribution of the GM to bone metabolism has attracted growing interest. Accumulating evidence indicates that the GM regulates bone homeostasis by shaping host immune responses and generating bioactive metabolites, particularly SCFAs. SCFAs inhibit osteoclast differentiation while enhancing osteoblast activity, thereby influencing BMD and biomechanical strength (8). In addition, SCFAs reinforce intestinal barrier function by upregulating tight-junction proteins (e.g., occludin), which may mitigate systemic lipopolysaccharide (LPS)–associated barrier disruption. SCFAs have also been reported to attenuate osteoarthritis (OA) progression, potentially by limiting inflammatory cell death in chondrocytes and restoring autophagy (37–39).

Beyond metabolite-mediated regulation, specific gut microbial taxa may exert more direct bone-protective effects along the gut–bone axis. Prevotella histicola (P. histicola), a commensal species enriched in healthy individuals (40), has been associated with protection against menopause-related bone loss in both human and animal studies and has been shown to attenuate bone loss in an ovariectomy (OVX)–induced osteoporosis model (41, 42). In addition, P. histicola suppressed disease manifestations in a humanized mouse model of inflammatory arthritis (43). Proposed mechanisms center on immunoinflammatory modulation, including restoration of the intestinal mucosal barrier, reduced intestinal permeability, diminished systemic inflammatory burden, and downstream suppression of pro-osteoclastogenic inflammatory mediators. In IBD, gut dysbiosis commonly co-occurs with chronic intestinal inflammation. This altered microbial state may exacerbate bone loss and increase the risk of disordered bone metabolism by amplifying systemic inflammatory signaling and disrupting the bone immune microenvironment (44).

### 3.3 Nutritional metabolism and disruptions in bone metabolism

Disordered nutritional metabolism is an important contributor to abnormalities in bone metabolism. In patients with IBD, nutritional insufficiency—particularly inadequate vitamin D and calcium intake or absorption—is common. Studies have demonstrated that vitamin D plays an essential role in calcium absorption and bone mineralization, yet vitamin D deficiency is highly prevalent among IBD patients due to impaired intestinal absorptive capacity (45). Insufficient vitamin D not only diminishes osteoblast activity but also suppresses bone formation, thereby increasing fracture risk and exacerbating inflammation-associated skeletal deterioration (46). Moreover, inadequate calcium intake triggers compensatory bone resorption to maintain serum calcium levels, further contributing to bone loss.

Obesity is closely associated with chronic inflammation, with elevated circulating levels of TNF-α, IL-1β, and IL-6 reported in both obese individuals and animal models (47); these mediators are produced largely by macrophages originating from adipose tissue. Beyond systemic inflammation, obesity may further amplify inflammatory responses via the “intestinal barrier–endotoxin” pathway: studies have shown that serum lipopolysaccharide (LPS) levels are markedly increased in patients with obesity-associated osteoarthritis (OA), potentially reflecting increased intestinal permeability that facilitates greater LPS translocation into the circulation and thereby exacerbates osteoarticular inflammation (48). Meanwhile, adipokines secreted by adipose tissue, including adiponectin and leptin, have been shown to modulate inflammatory immune responses in cartilage (49). Further studies have proposed that OA is not solely the result of mechanical wear; rather, metabolic dysregulation, chronic inflammation, and oxidative stress jointly drive its onset and progression. Accordingly, systemic nutritional interventions targeting the “metabolism–inflammation–oxidative stress” axis may serve as an important adjunct in the management of degenerative joint diseases (50), improving the joint microenvironment and clinical outcomes through foundational nutritional support, optimization of dietary structure, and modulation of metabolic inflammation.

### 3.4 The RANK/RANKL/OPG signaling pathway

The RANK/RANKL/OPG (receptor activator of nuclear factor-κB/receptor activator of nuclear factor-κB ligand/osteoprotegerin) signaling axis is a central regulator of bone remodeling, coordinating osteoblast–osteoclast coupling and intersecting with immune–inflammatory pathways (51). In an interleukin-2–deficient mouse model of spontaneous autoimmunity, increased RANKL production induces both spontaneous bone loss and colitis. Therapeutic modulation of RANKL–RANK signaling with exogenous recombinant osteoprotegerin (Fc-OPG) reverses skeletal abnormalities and significantly ameliorates colitis (52). Clinically, patients with IBD exhibit elevated plasma OPG levels and increased OPG release from inflamed colonic tissue, and OPG levels are inversely correlated with BMD. Collectively, these data implicate dysregulation of the RANK/RANKL/OPG axis in IBD-associated bone metabolic abnormalities (33). Moreover, activation of this pathway can synergize with pro-inflammatory cytokines, promoting excessive osteoclast activation and exacerbating bone resorption (53).

### 3.5 Dietary interventions and restoration of the gut microbiota

In efforts to improve bone health in patients with IBD, increasing attention has been directed toward dietary interventions aimed at restoring gut microbial homeostasis. Supplementation with probiotics, prebiotics, or specific dietary fibers—such as low-FODMAP components—has been shown to promote the colonization of beneficial microbial taxa and enhance the production of SCFAs. These metabolites suppress osteoclast activation while simultaneously supporting osteoblast function (8). Probiotic supplementation can partially restore gut microbial dysbiosis in dextran sulfate sodium (DSS)–induced colitis, improve intestinal barrier function, and thereby exert beneficial effects on bone metabolism by attenuating bone loss and reducing osteoporosis risk (54). Consistently, in animal models of osteoporosis, probiotics have been reported to increase bone mass and improve indices of bone turnover (55, 56). In addition, increasing the intake of calcium, vitamin D, and high-quality protein contributes to improved BMD and preservation of skeletal integrity (57). When combined with pharmacological therapy, such dietary strategies exert dual benefits: they directly supply essential substrates for bone formation while also modulating the GM and alleviating systemic inflammation, thereby enhancing the bone remodeling process.

Therefore, multidimensional intervention strategies that integrate GM modulation with nutritional optimization hold promise as essential components of bone health management in patients with IBD. Such approaches may offer novel therapeutic avenues for preventing and treating IBD-related osteoporosis.

## 4 Interactions between the gut and the brain

The interactions between the gut and the brain constitute a complex and dynamic bidirectional regulatory system collectively referred to as the GBA. This axis encompasses multiple pathways of information exchange—including neural signaling, endocrine regulation, and immune-mediated mechanisms—that together sustain functional communication between the gastrointestinal tract and the CNS (58, 59). Among these components, the enteric nervous system (ENS), often described as the “second brain,” plays a central role. Comprising approximately 200 million neurons embedded within the gastrointestinal wall, the ENS is capable of independently regulating intestinal motility, secretion, and local blood flow while maintaining close communication with the CNS (60). This highly intricate neural network not only modulates local gastrointestinal functions but also influences systemic physiological states and behavioral responses through its interactions with the CNS (61).

Within the GBA, the GM plays a central regulatory role. The trillions of microorganisms residing in the gastrointestinal tract produce a wide range of metabolites, including SCFAs, neurotransmitters, and other bioactive molecules, which influence brain function and behavior through multiple pathways (62, 63). For example, gut-derived SCFAs can cross the BBB or modulate cytokine levels to indirectly affect the central nervous system, thereby regulating neural circuits involved in emotion and cognition (58, 64). Some studies have proposed the concept of an “SCFAs–microglia pathway,” suggesting that SCFAs can regulate microglial activation either by directly entering the central nervous system and inhibiting epigenetic mechanisms such as histone deacetylases (HDACs) or by indirectly transmitting signals through peripheral immune cells via FFAR2/3-related pathways (65); in addition, SCFAs may improve the cerebral cellular microenvironment by suppressing excessive microglial activation, reducing inflammatory cytokine levels, and modulating mitochondrial metabolism to balance energy supply and immune function (66, 67). In addition, specific microbial taxa are capable of synthesizing neurotransmitters such as serotonin and γ-aminobutyric acid (GABA), both of which play critical roles in emotional stability, stress responses, and cognitive regulation (1) (As shown in **Table 1**). Collectively, these findings highlight that the GM profoundly shapes brain function and mental health through both metabolic and neurochemical signaling pathways.

**Table 1:** Gut microbiota-related mediators involved in gut–brain interactions with major mechanisms and linked outcomes.

Mediator	Main source	Proposed mechanisms of action	Main related outcomes
SCFAs	Gut microbiota-derived metabolites	(1) Direct effects by crossing the BBB; (2) indirect effects on the CNS by modulating immune cytokine levels; (3) enter the CNS and inhibit HDACs and other epigenetic mechanisms, or transmit signals indirectly via peripheral immune cells (FFAR2/3-related) to regulate microglial activation; (4) suppress excessive microglial activation; (5) regulate mitochondrial metabolism to balance energy supply and immune function	Influence emotion- and cognition-related neural circuits; reduce neuroinflammation; improve the brain cellular microenvironment; suggest potential intervention value for neurodegenerative diseases and mood/cognitive disorders
5-HT	Synthesis by enterochromaffin cells	Participate in immune responses and systemic signal regulation via the vagus nerve	Emotional stability, stress response, regulation of cognitive function
GABA	Production by *Lactobacillus*/*Bifidobacterium*	Modulate vagal regulation, immune inflammation, and changes in barrier function	Emotional stability, stress response, regulation of cognitive function
LPS	Bacterial products	(1) Damage to the intestinal barrier allows bacterial products to enter the bloodstream; (2) induce systemic inflammation; (3) promote neuroinflammatory responses and affect brain function	Promote neuroinflammation
Inflammatory factors	immune cells (e.g., macrophages)	(1) Disrupt the BBB; (2) amplify central neuroinflammation	Altered cognitive and emotional states; psychiatric and neurodegenerative diseases

Communication between the gut and the brain primarily relies on pathways such as the vagus nerve and the HPA axis (68). The vagus nerve serves as the principal conduit for transmitting changes in the gut environment—such as nutritional status and microbial composition—to the central nervous system, converting peripheral signals into neural responses that regulate autonomic functions and central plasticity (69). The HPA axis, through the release of corticotropin-releasing hormone (CRH) and cortisol, links psychological stress to metabolic and immune processes, thereby influencing gut barrier integrity and microbial homeostasis (70, 71). Additionally, the gut can indirectly affect brain function through immune-mediated pathways; for instance, gut-derived immune factors can induce neuroinflammation and consequently alter cognition and emotional states (72). Together, these mechanisms establish a multilayered bidirectional communication network through which gut health and neural function are tightly coupled under both physiological and pathological conditions.

Studies have demonstrated that GM dysbiosis is closely associated with a range of neurological and psychiatric disorders, including depression, anxiety, Alzheimer’s disease, Parkinson’s disease, and other neurodegenerative conditions (58, 59). Alterations in the composition and function of the GM can influence brain health through multiple mechanisms. On one hand, dysbiosis may increase intestinal permeability, allowing bacterial components such as lipopolysaccharides to enter the circulation, where they trigger systemic inflammation and promote neuroinflammatory responses. On the other hand, reductions in microbiota-derived metabolites (e.g., short-chain fatty acids, SCFAs) may weaken their regulatory effects on brain immune homeostasis, thereby impairing neuroplasticity and cognitive function (73, 74). Collectively, these findings suggest that maintaining gut microbial homeostasis not only helps prevent the onset of neurodegenerative diseases but may also hold therapeutic potential for improving mood disorders and cognitive impairment.

## 5 Immune mechanisms of the brain–bone–gut axis

Recent studies have established that the brain–bone–gut axis plays a critical role in the regulation of immune responses, with its core mechanisms involving multilayered interactions among the GM, the immune system, and the CNS (75–77). As a key regulatory component of this network, the GM can indirectly influence both brain and skeletal health by modulating systemic inflammation and immune cell function (78). The gut, serving as the primary interface between the host and its resident microorganisms, is particularly sensitive to alterations in microbial composition. Dysbiosis can increase intestinal permeability, allowing pro-inflammatory cytokines—such as tumor necrosis factor-α and interleukin-6—and bacterial metabolites to enter the circulation, thereby triggering neuroinflammation or disrupting bone metabolism (79). Microbial metabolites provide a mechanistic, molecular-level basis for communication along the gut–immune–brain/bone axis. SCFAs, representative products of dietary fiber fermentation by the GM, can modulate the magnitude and cytokine profile of inflammatory responses in neutrophils, macrophages, and T and B cells. These effects are mediated, at least in part, through inhibition of HDACs and activation of G protein–coupled receptors (e.g., GPR41/43 and GPR109A), thereby engaging key signaling nodes including NF-κB, MAPK, and mTOR. At the cellular level, butyrate can promote Foxp3 expression and induce colonic regulatory T-cell (Treg) differentiation (80), contributing to immune homeostasis and potentially reducing the burden of chronic inflammation (81).

In contrast, specific commensal bacteria can drive Th17 responses; for example, colonization of the small intestine by segmented filamentous bacterium (SFB) is sufficient to induce Th17 cells that produce IL-17 and IL-22 (82), indicating that the gut microecology can directly regulate the homeostatic tone of IL-17–related immune pathways. IL-17–related immunity can simultaneously span both the brain and bone: in the nervous system, Th17-associated cytokines (IL-17/IL-22) can act on blood–brain barrier endothelial cells and disrupt tight junctions, thereby promoting amplification of central inflammation and facilitating immune-cell entry into the CNS (83, 84). At the level of bone metabolism, Th17 cells have been shown to possess osteoclastogenic properties; IL-17/IL-23 are particularly critical during phases of bone destruction and can promote osteoclastogenesis and bone resorption by inducing pathways such as RANKL. Conversely, Tregs can inhibit osteoclast formation through CTLA-4–mediated direct cell–cell contact, thereby counteracting inflammation-associated bone loss at the “immune–bone” interface (85–87) and constituting a protective mechanism that balances Th17 activity. Therefore, alterations in the peripheral immune milieu may influence the osteoclast/osteoblast balance via the bone marrow immune microenvironment and may also modulate neuroinflammatory responses through neuroimmune pathways, linking gut immune dysregulation to pathological processes in both bone and brain.

At the level of the brain, the GM can influence immune responses within the CNS by regulating the synthesis of neurotransmitters and the production of neuroactive metabolites, mechanisms closely linked to the pathogenesis and progression of neuropsychiatric disorders such as depression and Alzheimer’s disease (88, 89). Meanwhile, the immune system also plays a central role in the regulation of bone metabolism. Findings from osteoimmunology indicate that pro-inflammatory cytokines secreted by immune cells—including T cells and macrophages—promote osteoclastogenesis and increase bone resorption, whereas anti-inflammatory cytokines enhance osteoblast activity and support bone formation (90).

Moreover, the vagus nerve, as the primary communication pathway between the gut and the brain, not only conveys information about gut health to the CNS but also indirectly influences bone metabolism by modulating inflammatory responses (91). This neuro-immune-skeletal interplay forms a fundamental physiological basis of the brain-bone-gut axis, resulting in a highly integrated coupling of gut health, immune homeostasis, and bone function under both physiological and pathological conditions.

## 6 Clinical evidence and disease associations

As is well known, the gut microbiome is essential for human health; it participates in the production of various gastrointestinal hormones, short-chain fatty acids, and vitamins, as well as in drug absorption and metabolism. Disruption of a healthy gut microbiome can lead to inflammation (92, 93). Patients with IBD frequently exhibit disturbances in the GM, which not only impair bone metabolism but also adversely affect psychological health through the GBA. Dysbiosis can elevate levels of pro-inflammatory cytokines, which in turn trigger emotional disorders and cognitive decline via neuro-immune pathways, while simultaneously exacerbating osteoporosis and osteoarthritis (94). Emerging evidence suggests that specific microbial taxa may have causal associations with bone diseases, potentially mediated through chronic stress, sleep disturbances, and cognitive impairment (95, 96). Systemic inflammation associated with IBD can also directly alter brain structure and function (97, 98). Pro-inflammatory cytokines can cross the BBB, induce neuroinflammation, worsen psychological symptoms, and reduce BMD (99, 100). Under chronic inflammatory conditions, the prevalence of anxiety and depression is markedly higher among IBD patients, which not only increases psychological burden but also influences bone metabolism through alterations in the HPA axis and autonomic nervous system, thereby elevating fracture risk (101).

In addition to neural pathways, the GM also directly regulates bone metabolism through the secretion of metabolites such as SCFAs (102, 103). This “gut–bone axis” plays a pivotal role in maintaining the dynamic balance of bone remodeling. Clinical and experimental studies indicate that interventions aimed at restoring gut microbial homeostasis—such as dietary modification, probiotic supplementation, or prebiotic intake—may simultaneously improve skeletal integrity and psychological well-being, dietary strategies such as the Mediterranean diet and high-fiber dietary patterns share a common biological rationale: by altering intestinal substrate availability and the luminal microenvironment, they can promote the enrichment of beneficial microbes and reshape intestinal inflammatory tone and immune homeostasis (104). In inflammatory bowel disease (IBD), these dietary modifications have been associated with improvements in disease activity and/or risk-related indicators; with respect to skeletal health, they correlate with lower fracture risk and enhanced mineral absorption; and clinical and cohort studies in neuroscience support potential benefits for depressive symptoms and cognitive function (105–108). Probiotics may also modulate the brain–bone–gut axis through multiple mechanisms, including attenuation of systemic inflammation, reinforcement of intestinal barrier integrity, modulation of microbial metabolites, and regulation of neurotransmitter and neuromodulator pathways, thereby influencing brain functional activity (109, 110). Consistent with these mechanisms, clinical studies have linked probiotic supplementation to improvements in cognitive performance and mood-related outcomes in selected populations (111). Notably, emotion-oriented psychobiotic interventions in randomized trials among patients with UC have been reported to reduce anxiety symptoms while also decreasing endoscopic inflammatory activity (112). Together, improvements in microbial composition and immune homeostasis may create a permissive microenvironment for maintaining bone metabolic equilibrium and, more broadly, support systemic health optimization (113).

Collectively, research on the brain–bone–gut axis provides a novel conceptual framework for understanding the interconnected relationships among mental health, skeletal diseases, and gut function. An expanding literature suggests that the “microbiota–immune–neuro/endocrine” interplay captured by the brain–bone–gut axis extends beyond IBD. In obesity and metabolic syndrome, gut-derived endotoxin–associated chronic low-grade inflammation may reciprocally reinforce dysregulated gut–brain neuroendocrine signaling (114, 115). In rheumatoid arthritis, gut dysbiosis and RANKL-mediated osteoclast activation have been linked to bone erosion and are frequently accompanied by chronic pain and affective comorbidities, including depression and anxiety (116–118). During aging, microbiota alterations are associated with bone loss and may also influence cognitive function via the GBA (119–121). Collectively, these observations support the broader relevance of this framework across diverse chronic diseases. Future investigations should focus on elucidating key signaling pathways and causal mechanisms across these systems and developing multimodal, microbiota-centered, personalized therapeutic strategies to enhance patient outcomes and support integrated prevention and management of multisystem disorders. Meanwhile, the potential of traditional medicine in interventions for bone-related diseases warrants attention. Taking knee osteoarthritis as an example, studies on Danggui Sini Decoction (122) provide preliminary evidence regarding its potential efficacy and safety; in the future, evidence-based research approaches such as systematic reviews and meta-analyses could be employed to further standardize the evaluation of its effectiveness and safety.

## 7 Conclusion

The brain–bone–gut axis provides an integrated framework for understanding the multisystem comorbidities involving the nervous system, skeletal system, and GM. The central nervous system regulates bone remodeling through neurotransmitters, neuropeptides, and hormones, while bone-derived hormones, in turn, influence cognition and emotional states. Meanwhile, the GM and its metabolites modulate mineral absorption, the RANK/RANKL/OPG signaling pathway, and systemic inflammation, thereby linking the “gut–bone axis” and the “GBA.” As a result, IBD, osteoporosis, osteoarthritis, depression, anxiety, and cognitive impairment often present with substantial comorbidity. Evidence from animal studies, clinical investigations, and Mendelian randomization analyses (i.e., using genetic variants associated with the exposure as instrumental variables for “naturally randomized” group assignment, thereby reducing confounding and enabling causal inference) collectively highlights the microbiota–immune–neuro network as a central therapeutic target. Microbiome-centered strategies, combined with nutritional optimization and physical activity interventions, hold the potential to simultaneously alleviate intestinal inflammation, mitigate bone loss, and improve neuropsychiatric symptoms. Future research should integrate multi-omics approaches with large-scale cohorts and prospective trials to delineate causal pathways involving key microbial taxa and metabolites and to clarify the molecular mechanisms underlying specific “microbe–metabolite–host” circuits. In parallel, clinical translation of microbiome-targeted interventions will require rigorous evaluation of safety, patient stratification strategies to support personalization, and verification of long-term effectiveness. With multidisciplinary collaboration, the brain–bone–gut axis framework can be incorporated into comprehensive chronic disease management and individualized treatment strategies. Finally, systematic reviews, meta-analyses, and other methodologically robust evidence-synthesis and quality-assessment efforts are needed to establish a translatable and generalizable clinical evidence base.
